# Rapid Identification of Bio-Molecules Applied for Detection of Biosecurity Agents Using Rolling Circle Amplification

**DOI:** 10.1371/journal.pone.0031068

**Published:** 2012-02-22

**Authors:** Jenny Göransson, Rongqin Ke, Rachel Yuan Nong, W. Mathias Howell, Anna Karman, Jan Grawé, Johan Stenberg, Malin Granberg, Magnus Elgh, David Herthnek, Per Wikström, Jonas Jarvius, Mats Nilsson

**Affiliations:** 1 the Rudbeck Laboratory, Department of Immunology, Genetics and Pathology, SciLifeLab, Uppsala University, Uppsala, Sweden; 2 Olink AB, Dag Hammarskjölds väg, Uppsala, Sweden; 3 Q-linea AB, Dag Hammarskjölds, Uppsala, Sweden; 4 Swedish Defense Research Agency, Umeå, Sweden; Deutsches Krebsforschungszentrum, Germany

## Abstract

Detection and identification of pathogens in environmental samples for biosecurity applications are challenging due to the strict requirements on specificity, sensitivity and time. We have developed a concept for quick, specific and sensitive pathogen identification in environmental samples. Target identification is realized by padlock- and proximity probing, and reacted probes are amplified by RCA (rolling-circle amplification). The individual RCA products are labeled by fluorescence and enumerated by an instrument, developed for sensitive and rapid digital analysis. The concept is demonstrated by identification of simili biowarfare agents for bacteria (*Escherichia coli* and *Pantoea agglomerans*) and spores (*Bacillus atrophaeus*) released in field.

## Introduction

Early recognition of hazardous biological materials is essential to all biodefence and biosafety strategies. Irrespective as to whether a release is deliberate, accidental or naturally occurring, early detection leads to improved intervention opportunities. Exposed individuals can be treated more quickly, and individuals at risk of exposure may receive prophylaxis in the form or vaccination or other medical treatment. Measures can also be taken to inhibit secondary spread of pathogenic agents and to contain the effects within a limited geographic range.

The demands on pathogen detection strategies for biodefence applications are high. It is of crucial importance to minimize false positive alarms to preserve the confidence for the detection system. At the same time, high sensitivity is equally important as false negative events will bring the full devastating effects on society that the system was installed to prevent. Moreover, pathogen detection and identification has to be done as rapidly as possible to maximize the effect of protective measures. Current identification approaches rely on genetic PCR-based analysis [1], [2], [3] or on immunoassay-based protein analysis [4], [5], [6]. The PCR-based assays are typically very sensitive, approximately 10 organisms or CFU per reaction in pure systems [7], [8], [9], and are known have a very high specificity as discriminatory single nucleotides can be targeted [10], [11]. However PCR assays tend to be sensitive to inhibitors in complex environmental samples [8], [12] and require trained personnel to interpret data. In addition, PCR-based applications are not very suitable for continuous sample processing such as required for surveillance of important sites in the community such as airports, subways, and other hubs of human communication. The current immunoassays are subjected to limitations in specificity, due to cross-reactivity with non-pathogenic naturally occurring close relatives [13], [14], and sensitivity when analysing samples from environmental matrices [15], [16] and are therefore problematic to use in biowarfare applications.

We have developed a system for environmental monitoring of pathogens based on homogenous amplified single-molecule detection, or RCA [17], [18], [19], [20]. The system employs padlock probes for genetic analysis and proximity ligation assay (PLA) for sensitive and specific protein detection [21], [22], [23], [24], [25], [26], [27], [28]. Padlock probes are linear oligonuleotides containing two target-complementary end-sequences, designed such that the probes become circularized through ligation upon hybridization to a specific target DNA sequence. Proximity ligation assay employs two antibodies equipped with oligonucleotides that template a DNA circularization reaction upon proximal coincident binding to the same target molecule. We have developed these molecular probing mechanisms to achieve faster detection with retained sensitivity. To achieve this we have developed a protocol for on-bead padlock probing and rolling circle amplification (RCA). The improved reaction kinetics of the on-bead probing and amplification protocol was enabled by (i) increasing the concentration of padlock probes drastically, and by (ii) including a second, biotinylated probe that captures template sequences to a streptavidin-coated solid support. Thereby any unreacted padlock probes could be removed to avoid their negative interference with the subsequent RCA. Reacted probes are amplified through two cycles of RCA, the first on the solid-phase and the second in solution, using the circle-to-circle amplification (C2CA) mechanism [29]. The resulting RCA products (RCPs) are then tagged with fluorescence, and thereby become readily visible over background fluorescence. Detection of the RCPs is realized by an instrument equipped with a high-performance fluorescence detector. Thresholding of the registered files enables counting of the individual RCPs through binarization of the data, reflected as signals, ‘1’, in contrast to background fluorescence falling below threshold, referred to as ‘0’ (Figure S1). This technique is analogous to digital PCR, in which fluorescence-labelled amplicon clusters deriving from individual templates are enumerated, but our approach does not require compartmentalization in water-in-oil emulsion or massively parallel microwell plates [30], [31]. The total reaction time of the protocol is 30 min with retained LOD. This study furthermore presents stream-lining of the padlock probe and PLA protocols, to present a protocol that comprise several identical reaction mixtures and that should lend itself to automation. To summarize, the protocols and instrumentation presented herein represents a substantial improvement from previous protocols and instrumentation, and pose a unique and promising opportunity for quick and sensitive detection of pathogens Furthermore, we herein for the first time apply PLA with a digital readout for an analyte in solution.

## Materials and Methods

### Preparation of target agents

The target bacteria were prepared by cultivating *Escherichia coli* (EC) strain TOP10 (Invitrogen, Paisley, UK) and *Pantoea agglomerans* (PA) strain ATCC 33243 on BAB plates overnight (Merck, Darmstadt, Germany). The colonies were picked and diluted in NaCl buffer to a concentration of 10^7^ CFU/ml. A dry preparation of *Bacillus atrophaeus* spores was dissolved in NaCl buffer to a concentration of 10^8^ spores/ml. We will use the acronym BG for these spores, since they were previously known as *Bacillus globigii* and BG is still often used to distinguish them from *Bacillus anthracis*, usually abbreviated BA.

### Target agent dissemination and air sampling

The dissemination of these agents was done in a forest environment to mimic a realistic scenario where the target target agent is sampled together with a natural microbiological background. The samples were taken at the Swedish Armed Forces test range with duly permission to disseminate this type of biological agent simulants. The air sampler ASAP (Thermo Scientific, NY, USA) was placed on a platform with dissemination equipment in close proximity in such a way that the majority of the aerosolized target agents were collected (Figure S2). The dissemination was done with a flow of 1 ml/min. It was approximated that everything that were dispersed was sampled by the air sampler. Every sampling occasion consisted of 15 min air sampling with varying duration of the disseminations ranging from 3 seconds to 8 min. The ASAP sampled the air at a rate of 200 l/min. For example, 3 seconds of target agent dispersal was followed by 15 min sampling of only natural background air.

The ASAP filters containing the target agents were further processed within two hours from the dissemination. Each filter was removed from the ASAP cartridge and placed in a 1.5 ml-tube. The subsequent sample preparation was done along two different routes depending on the target agent.

### Sample preparation

#### Preparation of DNA

For the EC and PA samples, 200 µl of L6-buffer (10) was added to each tube followed by 5 min incubating at 56°C. Five hundred microliters of binding buffer (QuickPick® gDNA kit, Bio-Nobile Oy, Parainen, Finland) was added followed by squeezing out the liquid from the filter to a new 1.5-ml tube. Magnetic beads (8 µl) were added and followed by manually shaking and incubation for 5 min at room temperature. The magnetic beads, now with bound nucleic acids, were transferred with a PickPen® (Bio-Nobile Oy, Parainen, Finland) and washed three times according to the QuickPick SML gDNA protocol. Finally, the DNA was eluted from the beads in 60 µl H_2_O after shaking for 5 min at room temperature. The magnetic beads were then discarded with the PickPen® and the remaining nucleic acid-containing liquid was ready for the padlock probing reaction.

#### Preparation of protein

For the BG spores samples, 500 µl of PBS buffer was added to each filter-containing tube. Before discarding the filter, the liquid was squeezed out and the remaining liquid containing the spores was ready for the PLA.

### Padlock probe assay

Padlock probes and target capture probes were ordered from Integrated DNA Technologies (Munich, Germany). The probes were designed to detect motifs of 16S rDNA of EC and PA, respectively. The sequences of the padlock probes and capture probes are listed in Table 1.

**Table 1:** Sequences of the oligonucleotides used in the study. Underlined nucleotides are locked nucleic acids [17].

Name	5′ modification	Sequence	Function
Pd_EC	Phosphate	5′-TTAATACCTTTGCTCATTGACAGAGTGTATGCAGCTCCTCAGTATAGTCGATAGTCACGGCTACTTTTGGAAGGGAGTAAAG	*E. coli padlock* probe
Pd_PA	Phosphate	5′AAAGTGCGTCGTAGTCAGTCGATAGTCACGGCTACTAGAGTGTATGCAGCTCCTCAGTACAAGCGGACCTCAC	*P. agglomerans* padlock probe
CP_EC	Biotin	5′-CTCTCTCTCTCTCTCTCTCTCTCTCTGAAGAAGCACCGGCTAACTCCGTGCCAGCAGCCGCGGTAA	*E. coli* capture probe
CP_PA	Biotin	5′-TCTCTCTCTCTAAGCTTCCTCTCTCTCCGTGAAGTCGGAATCGCTAGTAATCGTGGATC	*P. agglomerans* capture probe
Arm_1	Thiol	5′-AAAAAAAAAAGACGCTAATAGTTAAGACGCTTUUU	PLA arm
Arm_2	Thiol	5′-AAAAAAAAAATATGACAGAACTAGACACTCTT	PLA arm
PLA_BB	Phosphate	5′P-TATTAGCGTCCAGTGAATGCGAGTCCGTCTAAGAGTGTATGCAGCTCCTCAGTATCAAGAGTGTCTA	PLA connector oligonucleotides
PLA_SL	Phosphate	5′P-GTTCTGTCATATTTAAGCGTCTTAA	PLA connector oligonucleotides
RO+	None	5′-GTGTATGCAGCTCCTCAGTA	Restriction digestion oligonucleotides
RO−	None	5′-TACTGAGGAGCTGCATACAC	RCA primer
DO_1	Cy3	5′Cy3-AGTAGCCGTGTTC*UUUU*	Detection oligonucleotides
DO_2	Cy3	5′Cy3-ACTATCGACTTTC*UUUU*	Detection oligonucleotides
DO_EC	Cy3	5′-TCGTGTAAGACACTATCCAC*UUUU*-3′	Detection oligonucleotides for E.coli in selectivity experiment
DO_PA	Alexa Fluor 488	5′-ATCGGCCTGTAATCGGATCG*UUUA*-3′	Detection oligonucleotides for *P. agglomerans* in selectivity experiment
NRP	Alexa Fluor 488	5′ - TTTTTTTTTTTTTTTT*UUUU*- 3′	Noise reduction probe

Nucleotides in italic style are 2′OMe-RNA. The NRP is furthermore labeled by biotin in the 3′end.

The hybridization of capture probes and ligation of padlock probes to the target DNA were performed simultaneously, and was achieved by incubating fragmented and denatured genomic DNA in 20 mM Tris-HCl (pH 8.3), 25 mM KCl, 10 mM MgCl_2_, 0.5 mM NAD, 0.01% Triton® X-100, 100 nM padlock probe, 50 nM capture probe, 0.2 µg/µl BSA (New England Biolabs, MA, USA), and 250 mU/µl Ampligase (Epicentre Biotechnologies, WI, USA) at 55°C for 5 min. The target DNA along with reacted padlock probes were captured onto magnetic particles via the biotinylated capture probes. This was achieved by adding 50 µg Dynabeads MyOneTM Streptavidin T1 beads (Invitrogen) to the hybridization/ligation reaction and incubating the sample at room temperature for 3 min. Excess probes were eliminated by washing once with 100 µl washing buffer containing 5 mM Tris-HCl (pH 7.5), 5 mM EDTA, 1 M NaCl, and 0.1% Tween-20. The elimination of excess linear padlock probes is necessary, since these would otherwise interfere negatively with the subsequent RCA reaction.

The selectivity experiment was performed similarly, but included two padlock probes and two capture probes for targeting of either EC or PA. Furthermore, the duration of the RCA reactions was 20 minutes each. The RCPs were labeled in a separate labeling step by adding 0.1% Tween-20, 0.5 M NaCl, 20 mM EDTA, 20 mM Tris-HCl (pH 8), 5 nM DO_EC (Alexa-488) and 5 nM DO_PA (Cy3) to a final volume of 50 µl. The hybridization reaction was performed at 70°C for 2 minutes and at 55°C for 15 minutes.

Amplification of reacted probes was performed by C2CA as described below.

### Solid phase PLA

Polyclonal goat anti-BG spore antibody was provided by FOI Sweden. The biotinylation of anti-BG spore antibody, and conjugation of PLA oligonucleotides to prepare PLA probes were performed by Olink Bioscience (Uppsala, Sweden). The sequences of the PLA oligonucleotides are listed in Table 1.

One mg of Dynabeads MyOneTM Streptavidin T1 beads was prepared by washing twice with PLA washing buffer (1×PBS, 0.05% Tween-20) and was thereafter resuspended in 100 µl storage buffer (0.1% BSA, 1×PBS, 0.01% NaN_3_), and mixed with 100 µl 100 nM biotinylated goat anti-BG spores antibody in storage buffer. The coupling reaction was incubated at room temperature for 2 hours. The antibody-equipped beads were thereafter washed 3 times in washing buffer and reconstituted with 200 µl storage buffer. The resultant beads were stored at 4°C until used.

Prior to the PLA reaction, the bead storage buffer was removed and replaced by an equal volume of PLA buffer (1 mM D-biotin (Invitrogen), 0.1% BSA (Sigma-Aldrich), 0.05% Tween-20, 100 nM goat IgG (Sigma-Aldrich), 0.1 µg/µl salmon sperm DNA (Invitrogen), 5 mM EDTA, 1×PBS) containing 50 nM of each PLA probe. The BG spore dilution series was prepared by diluting the BG spore stock in PLA buffer. The environmental samples were diluted 10 times by PLA buffer prior to use. Fourty-five microliters of spore dilution series or environmental samples were added to each well, followed by addition of 5 µl goat anti-BG antibody coated magnetic beads and PLA probes mixture. The solution was mixed gently by pipetting and incubated at room temperature for 3 min, followed by removal of the supernatant and washing with PLA washing buffer three times. Fifty microliters of ligation mixture (20 mM Tris-HCl (pH 8.3), 25 mM KCl, 10 mM MgCl_2_, 5 mM NAD, and 0.1% Triton® X-100, 100 nM of each connector oligonuclieotide, 2.5 U ampligase (Epicentre Biotech)) was added to each reaction, and the microparticles were resuspended by gentle vortex. The ligation reaction was then incubated at 50°C for 5 min, followed by washing to eliminate excess PLA connector oligonucleotides. Amplification of reacted probes was performed by C2CA as described below.

### Circle-to-circle amplification (C2CA)

Reacted probes were amplified by C2CA, which includes serial enzymatic reactions starting with RCA. The RCA reaction was initiated by the addition of 20 µl ligation mixture containing 1× phi29 DNA polymerase buffer (Fermentas, Lithuania; 33 mM Tris-acetate (pH 7.9 at 37°C), 10 mM Mg-acetate, 66 mM K-acetate, 0.1% (v/v) Tween-20, 1 mM DTT), 100 µM dNTPs, 0.2 µg/µl BSA, 25 nM primer, and 100 mU/µl phi29 DNA polymerase. The reaction was incubated at 37°C for 11 min, and inactivated at 65°C for 1 min. The RCA products were digested at 37°C for 1 min by the addition of 3 units of AluI (New England Biolabs), 600 nM replication oligonucleotide, 0.2 µg/µl BSA in 1× phi29 DNA polymerase buffer, and the reaction was terminated at 65°C for 1 min. Ligation, amplification and labeling reactions were performed by the addition of a mixture containing 1.36 mM ATP, 100 µM dNTPs, 0.2 µg/µl BSA, 10 nM detection probe 1 (DO_1 Cy3), 10 nM detection probe 2 (DO_2 Cy3), 10 nM noise reduction probe (NRP Alexa-488), 28 mU/µl T4 DNA ligase and 120 mU/µl phi29 DNA polymerase in 1× phi29 DNA polymerase buffer to a final volume of 50 µl. The reactions were incubated at 37°C for 7 min, and terminated at 65°C for 1 min. The RCPs were now ready for analysis.

### Detection of labeled RCPs

Solutions containing the labeled RCPs were analyzed using a dedicated high-speed fluorescence detection instrument (Text S1). Briefly, the sample solution is pushed through a flow cell, a line across which is illuminated by lasers of multiple wavelengths. Fluorescent light of wavelength ranges corresponding to the emission spectra of the fluorescent labels is collected by CCD line detectors and the generated images are subjected to computer image analysis to identify and determine the number of RCPs containing each of the labels (Figure S6).

## Results

The approach for detection of bio-molecules presented in Jarvius *et al* [20] has been completely revised in order to allow rapid and specific detection with a low limit of detection (LOD). In the Jarvius *et al* version, the major time of the protocol is spent on the ligation reaction, reflecting that this reaction is inefficient. The key feature for improving the ligation reaction efficiency is to increase the hybridization kinetics by increasing the probe concentration. This action however requires actions to eliminate excess probe molecules prior to the amplification reactions, since these have been shown to interfere with amplification step [20], [29], [32]. The introduction of a solid-phase to which the target along with the reacted probes are bound, enables elimination of unreacted probes through a washing step.

**Figure 1:**
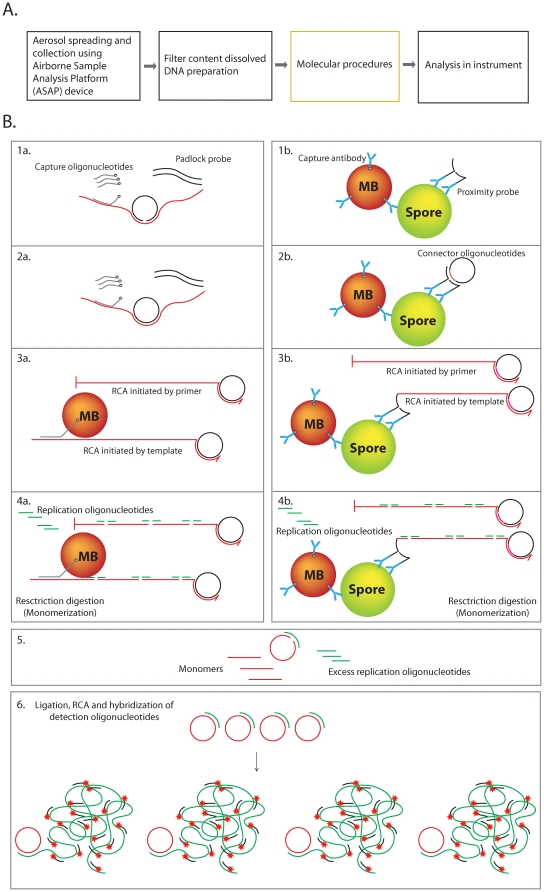
Detection scheme of the bio-monitoring system. A) Collection of environmental samples is realized using the Airborne Sample Analysis Platform (ASAP) equipment. Aerosolized particulates stick to a filter, and the content is extracted and analyzed. In contrast to protein detection, detection of nucleic acids requires preparation of the filter content prior to exposure to the molecular procedures. The molecular procedures detect the target molecules using dedicated probes and reacted probes are then amplified. The amplification products are analyzed using a dedicated prototype instrument. B) The molecular procedures of DNA (left) and protein (right) detection. Detection of nucleic acids is achieved by padlock probes that are specifically circularized if correctly hybridized to the correct target in the presence of DNA ligase. Padlock and capture probes are added to the samples along with DNA ligase (5 min). Reacted padlock probes are captured on magnetic beads and excess probes are eliminated by washing (3 min). Detection of proteins is initiated by capture of the target protein using magnetic beads equipped with antibodies. The addition of a pair of PLA probes, which are antibodies with attached oligonucleotides (3 min), forms a DNA circle guided by two connector oligonucletides, and a DNA ligase (5 min). Unreacted probes are eliminated by washing the circles, and from this step the magnetic beads are treated identically in both the genetic and PLA assays. A first RCA is initiated either by an extra primer or the target itself on the beads to replicate the DNA circles (11 min). The products are then restriction digested (2 min), and the monomers are collected. The monomers can then bind head-to-tail to the excess amount of replication oligonucleotides and formed new DNA circles. The new circles are amplified and labeled with fluorescence-tagged detection probes. The ligation, amplification and labeling are performed in one reaction (8 min). The RCPs are therefore ready for analysis in the detection instrument.

Target molecules can be either DNA or proteins, and input samples may derive from content extracted from air filters. Special notice has been taken to enable amplification of both DNA and protein samples using the same reaction mixtures. A dedicated prototype instrument has been developed and the products have been analyzed using this equipment, allowing improved sensitivity. The overall approach is outlined in Figure 1A. The molecular detection and amplification procedure is illustrated in Figure 1B.

### Genetic detection

Briefly, detection of nucleic acids is accomplished through denaturing the target DNA, which is thereafter probed by a biotinylated capture probe and a padlock probe. The capture probe enables reacted padlock probes to be captured to streptavidin-coated magnetic beads *via* sandwich hybridization to the target DNA strand. The biotinylated capture probe hybridizes upstream of the padlock probe target sequence (Figure 1B, left). The total time of the protocol has been reduced by twelve times from 6 hours to only 30 minutes with retained sensitivity (3). The incubation time of the padlock probe ligation step has been drastically reduced from three hours to only five minutes by increasing the concentration of padlock probes significantly. The use of a solid-phase for capture of the target molecules enables facile elimination of unreacted probes through washing, as probes at this high concentration would otherwise interfere with the following RCA.

### Protein detection

A protocol for quick, digital PLA has been developed and applied for spore detection, wherein the spores are captured by antibodies immobilized on the magnetic beads. A pair of PLA probes is applied to the sample. Upon proximal binding of the PLA probes, DNA circles are formed guided by two connector oligonucleotides and a DNA ligase (Figure 1B, right). The use of magnetic beads enables washing to remove excess reagents that could interfere with subsequent reaction steps, and also allows the use of a high concentration of beads and PLA probes, thereby improving the kinetics of the assay permitting a decrease in the reaction time from one hour to five minutes. This is also the first digitalized PLA for an analyte in solution.

### Signal amplification

**Figure 2:**
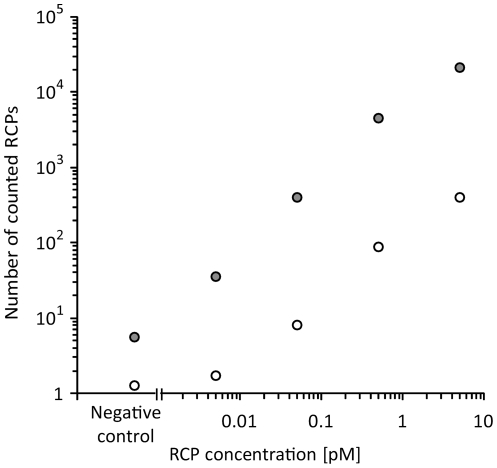
Comparison between confocal microscopy and the dedicated instrument for detection of RCPs. The quantitative response of the same dilution series of EC DNA was measured using the dedicated instrument as well as the confocal setup used in Jarvius *et al* 2006. Filled symbols: dedicated instrument, open symbols: Zeiss 510 Meta confocal microscope.

The presence of target DNA and proteins results in the formation of circularized DNA molecules by the probing mechanisms described. From this point in the procedure, both protein and DNA targets are processed using the same protocol. Reacted probes are amplified on the beads by RCA to produce long single-stranded concatemers (RCPs). Highly sensitive detection is achieved by C2CA [20], [29], wherein the number of DNA circles is amplified as follows (detailed in Figure 1): The RCPs produced in the first RCA are digested into monomers by a restriction enzyme. The linear monomers are then circularized through ligation, forming new circles of an amount that is proportional to the RCA time. The circles act as templates for a second RCA to generate RCPs that are labelled via hybridization of fluorescence-modified oligonucleotides during the amplification process and thus immediately ready for detection. The new detection instrument (for detailed description see Text S1) has a limit of detection that is at least 10 times better than to the previously used confocal microscope set-up (Figure 2). A number of restriction endonucleases were screened in search of enzymes that could cut rapidly (1 min) and be heat inactivated rapidly (1 min) (Table S1). We found *Alu*l to have the desired properties, and by using this enzyme rather than *Rsa*I as used in the previous protocol [20], the cleavage and inactivation steps could be completed within 2 min, saving an additional 18 min (Figure S4 and Figure S5). In the current protocol, we have reduced the number of necessary pipetting steps by combining the ligation, RCA and labelling into a single reaction. The incubation time of this second RCA influences the intensity of the labelled RCPs. The longer the amplification time, the more repeats of the detection sequence are incorporated into each RCP thus increasing the number of hybridization sites for the fluorescence-labelled detection probes.

The time of the second RCA was reduced by 53 min. Each RCP then contains around 150 copies, each labelled with two short detection probes. Four nucleotides of the detection probes were exchanged for LNA bases, which increased the melting temperature of the probes. Furthermore, the detection probes were blocked with 3′-mismatched 2′O-Me-RNA residues to protect the probes from 3′ exonucleolytic degradation, as well as extension by the phi29 DNA polymerase [33]. These changes of the detection probes enabled labelling of the RCPs during the RCA. Any magnetic beads still present in the solution can introduce background signals since they are of similar size and have prominent auto-fluorescence. To eliminate this source of background signals, a noise reduction probe (NRP) has been introduced, reducing the risk of false positives signals in the detector. The NRP is equipped with biotin in the 3′end and AlexaFluor 488 in the 5′end and efficiently labels beads in the solution. The RCPs are detected in the Cy3 channel and dual-labeled signals are rejected as false signals.

### Sensitivity

**Figure 3:**
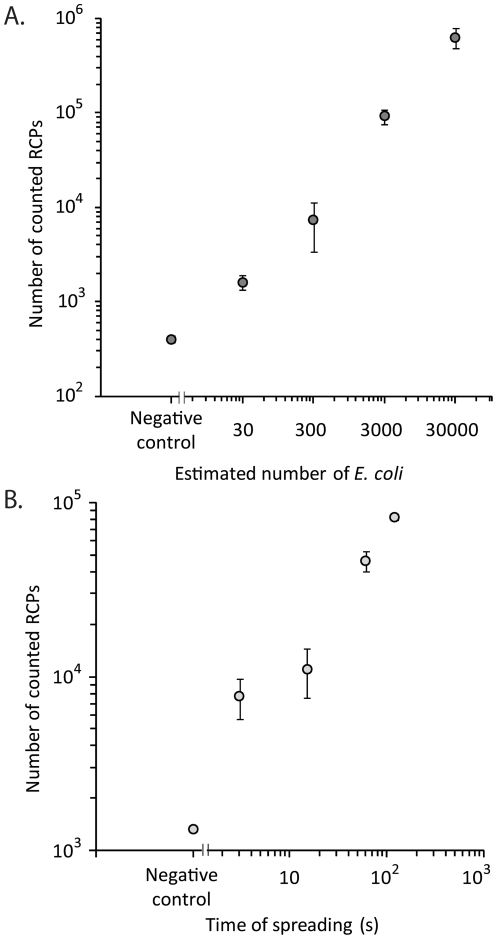
Identification of bacteria using padlock probe-based genetic analysis. A) Quantitative response measured in a dilution series of EC DNA. Error bars, ±1 s.d.; n = 3. B) Detection and identification of EC bacteria in environmental samples of air. All air samples were collected for 15 min, using the indicated spreading times for release of bacteria. Error bars, ±1 s.d.; n = 3.

**Figure 4:**
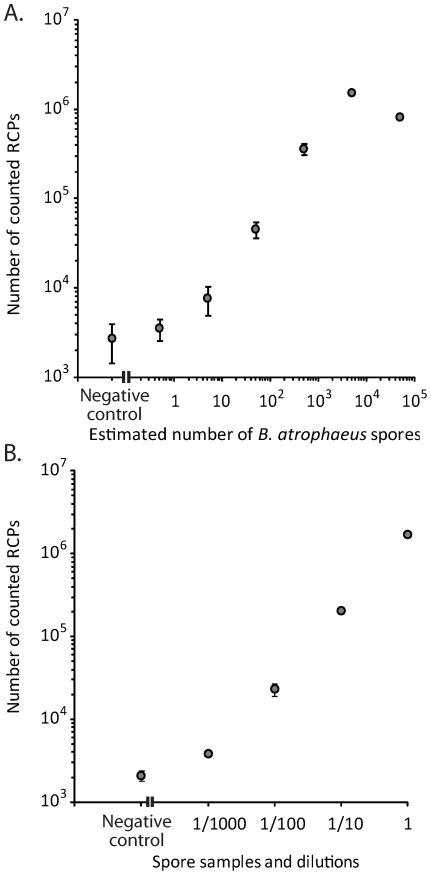
Detection of BG spores using PLA. A) Quantitative response measured in a dilution series of BG spores. Error bars, ±1 s.d.; n = 3. B) Detection and identification of BG spores in environmental samples of air. A positive sample was diluted in a background sample collected for 15 min. Error bars, ±1 s.d.; n = 2.

Genetic assays were designed for detection of the bacteria *Escherichia coli* (EC) and *Pantoea agglomerans* (PA) and a protein detection assay was set up for BG spores. The sensitivity of the assay was evaluated on dilution series of purified material. The LOD for the genetic assay is less than 30 bacteria using a combined processing time of 30 min, excluding DNA purification (Figure 3A). This LOD was similar to the one achieved by Q-PCR analysis of the same samples (Figure S3). The corresponding sensitivity for spore detection is 5 spores in 50 µl samples (Figure 4A).

### Specificity

**Figure 5:**
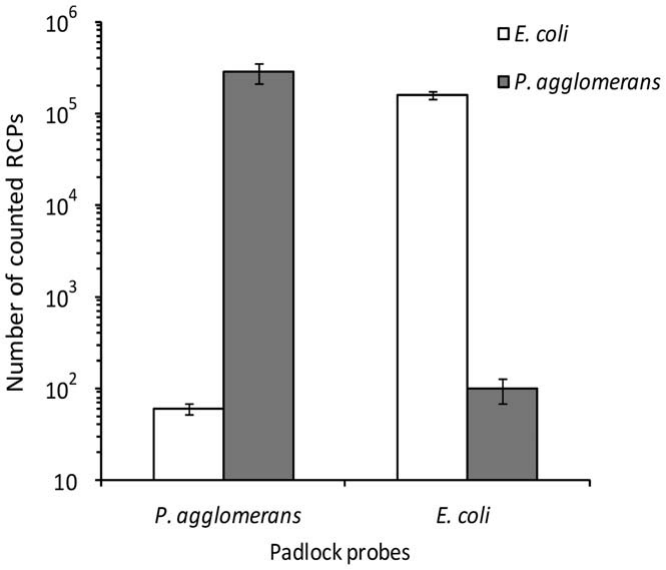
Discrimination of EC and PA bacteria based on a single nucleotide difference in the target DNA sequence in two samples containing either 1 ng EC genome or PA genome (approximately 300 000 copies), assayed with probes specific for EC and PA genomes in duplex reactions. Error bars, ±1 s.d.; n = 2.

The padlock probe and proximity ligation assays are highly specific probing assays that rely on dual or more recognition events on the same target as well as the action of a DNA ligase [21], [22]. This multi-recognition system enables highly specific detection of bio-molecules, reducing false positive signals caused by binding of detection reagents to non-target molecules. DNA ligation reactions can under standard reaction conditions robustly discriminate between target sequences that differs at single nucleotide positions [34], [35]. The specificity of the assays was investigated on environmental samples of air collected in the forest outside Umeå in Sweden. EC and PA bacteria as well as BG spores spread in aerosols and air was sampled using Airborne Sample Analysis Platform (ASAP Model 2800) equipment, with an approximate recovery of 0.2‰ of the DNA and 3‰ of the spores. For the padlock probe-based genetic assays, DNA was extracted using a simplified version of the QuickPick® gDNA kit (Bio-Nobile Oy, Parainen, Finland). For the proximity probe based spore detection assays, no purification or extraction was done prior to analysis. The experiment shows both assays to be selective towards the natural background of forest air, since negative control samples collected without previous spreading of spores and bacteria did not give a significant response (Figures 3B and 4B). Furthermore, data agreed well with Q-PCR control experiments (Figure S3). For the genetic analysis the specificity was further demonstrated by discriminating between the two bacterial species based on a single-nucleotide difference between the target DNA sequences of EC and PA. The two padlock probes used in this reaction differ by a single nucleotide at the 3′end, but are equipped with two completely different tag sequences that are used as target sequences for the two differentially labelled detection probes used to label the RCPs. The two bacterial species are clearly distinguished, with a selectivity of at least a factor of 10^3^ (Figure 5).

## Discussion

The bio-monitoring approach presented here is based on the homogeneous amplified single-molecule detection technique described by Jarvius *et al* [20]. A protocol for molecular probing and amplification which is quick and amenable to automate has been developed and a dedicated prototype instrument has been constructed. In this study the focus has been bio-security applications, however, the system is generic and could be useful for several applications, including water and food quality testing, and high-precision digital analysis for research and diagnostic applications.

The time of the molecular probing and amplification procedures have been reduced from about six hours to 30 min with retained sensitivity. The number of processing steps has also been reduced from six to four.

The analysis of RCPs was previously performed using a confocal microscope as fluorescence detector and a separated pump for driving the microfluidic system. We have developed a dedicated detection instrument which is rapid, and with an at least tenfold increased sensitivity. Furthermore, incorporation of multiple lasers and detectors in the instrument enabled, for example, simultaneous screening of PA and EC.

The approach developed in this study enables digital detection of both DNA and protein in the same analysis platform. This versatile feature enables multiple detection strategies for challenging agents, such as spores, where the DNA can be difficult to address directly. We applied the assays for detection of EC and PA on a DNA-level with a LOD of approximately 30 bacteria. The sensitivity is relevant for detection of several hazardous agents [36], like *Bacillus anthracis* and *Yersinia pestis*. Compared to Q-PCR, as discussed in more detail in Jarvius et al [20], the LOD is similar. The multiplexing ability is better since there is no spectral cross-talk between the discrete signals in this single-molecule approach. The linear dynamic range is worse in the current set-up, but can be extended. Also, the approach is less sensitive to inhibitory substances in the sample matrix [20]. BG spores were detected with a LOD of around 5 spores in 50 µl sample. PLA has previously been readout by Q-PCR and applied for detection of a wide range of protein analytes in both double-binder homogenous assays and triple-binder solid-phase assays [22], [23], [25], [26], [27], [28], but this is the first time PLA has been applied for digital analysis of analytes in solution. The limit of detection of PLA-based immunoassays is typically at least ten times better than standard sandwich ELISA assays [22], [23], [28].

Detection of specific biological materials present in the environment with naturally occurring close relatives of the target organism, places high demands on assay specificity. We show that our approach can be used to analyze realistic environmental samples containing the full complexity of biomaterials in the air of a Swedish forest in the summer.

The protocols and the instrument together constitute a universal system for quick, sensitive and specific detection of bio-molecules (proteins or nucleic acids), which generates digital data that is easy to interpret. The random access mode of operation makes it suitable for continuous sampling at a rate of up to one sample per minute. The system offers rapid and specific detection, combined with a low LOD, and constitutes a powerful method for early recognition of hazardous biological materials.

## Supporting Information

Figure S1 **A part of a scanned image showing the RCPs.** Each RCP is visualized as a bright dot, and is therefore counted as ‘1’. The figure is not drawn to scale.
(TIF)

Figure S2 **A picture showing how the dissemination equipment and the air sampling system work.**
(TIF)

Figure S3 **Detection of** ***E coli*** **genome by quantitative PCR.** A) A dilution series of genomic DNA isolated from *E coli* was analyzed by quantitative PCR. B) Prepared samples from spreading by the ASAP air sampler detected by quantitative PCR. The negative control sample is water. The standard deviations are from triplicate samples.
(TIF)

Figure S4 **Lambda DNA Digestion products with and without preceding incubation at 65°C.** For both lanes 2 in A and both lanes 10 in B: 1 kb ladder. Both lanes 3 in A: undigested Lambda DNA. Under each enzyme legend in A and B, the first lane contains DNA digested for 5 min after incubation at 65°C 5 min and the second lane contains DNA digested for 5 min after incubation at RT.
(TIF)

Figure S5 **Lambda DNA Digestion products with and without preceding incubation at 65°C using different incubation periods.** First and last lane in both rows: 1 kb ladder. Lane 2, both rows: undigested Lambda DNA. Under each enzyme legend, the first three lanes contain DNA digested for 5 min after incubation at 65°C for 1, 2 and 4 min respectively. The following three lanes contain DNA digested for 1, 2 and 4 min respectively, after incubation at RT.
(TIF)

Figure S6 **Schematic description of the optical set-up of the detection instrument.** A) Optical pathway fluorescence excitation schematics. a) 640 nm laser, b) 532 nm laser, c) 491 nm laser, d) beam expenders, e) laser mirrors, f) 532/640 nm laser beam combiner, g) 491/532/640 nm laser beam combiner, h) beam shaping optics, i) triple-laser pass dichroic mirror, j) objective, and k) flow channel. B) Optical pathway fluorescence emission schematics. a) CCD line detector, b) 648 LP dichroic mirror, c) 550 LP dichroic mirror, d) tube lens, e) beam pickoff, f) beam monitoring tube lens, g) beam monitoring CCD camera, h) notch filter 532 nm, i) laser pass dichroic mirror, j) objective, k) detection channel, l) beam monitoring CCD camera filter (590/60), m)525/50 band-pass filter, n) 590/60 band-pass filter, and o) 690/60 bandpass filter.
(TIF)

Table S1 **Observed digestion and inactivation properties of the tested restriction enzymes.**
(DOCX)

Text S1 **Supporting materials and methods.**
(DOCX)
