# Supplementary figures and images for: Rapid Identification of Bio-Molecules Applied for Detection of Biosecurity Agents Using Rolling Circle Amplification
Source: PLoS One. 2012 Feb 22;7(2):e31068. doi: 10.1371/journal.pone.0031068 (PMC3285169; doi:10.1371/journal.pone.0031068)

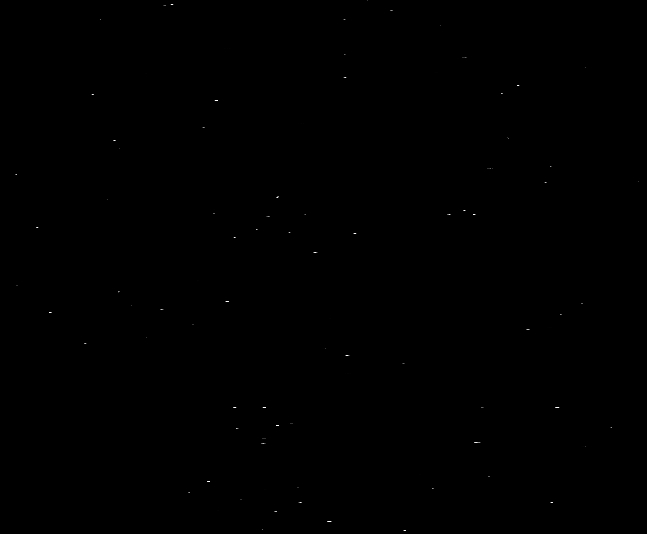

Supplement: Figure S1 — A part of a scanned image showing the RCPs. Each RCP is visualized as a bright dot, and is therefore counted as ‘1’. The figure is not drawn to scale. (TIF) [file pone.0031068.s001.tif]

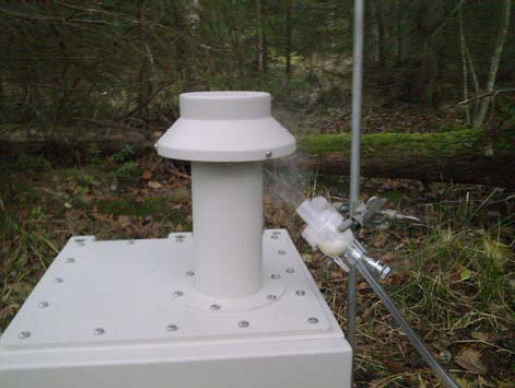

Supplement: Figure S2 — A picture showing how the dissemination equipment and the air sampling system work. (TIF) [file pone.0031068.s002.tif]

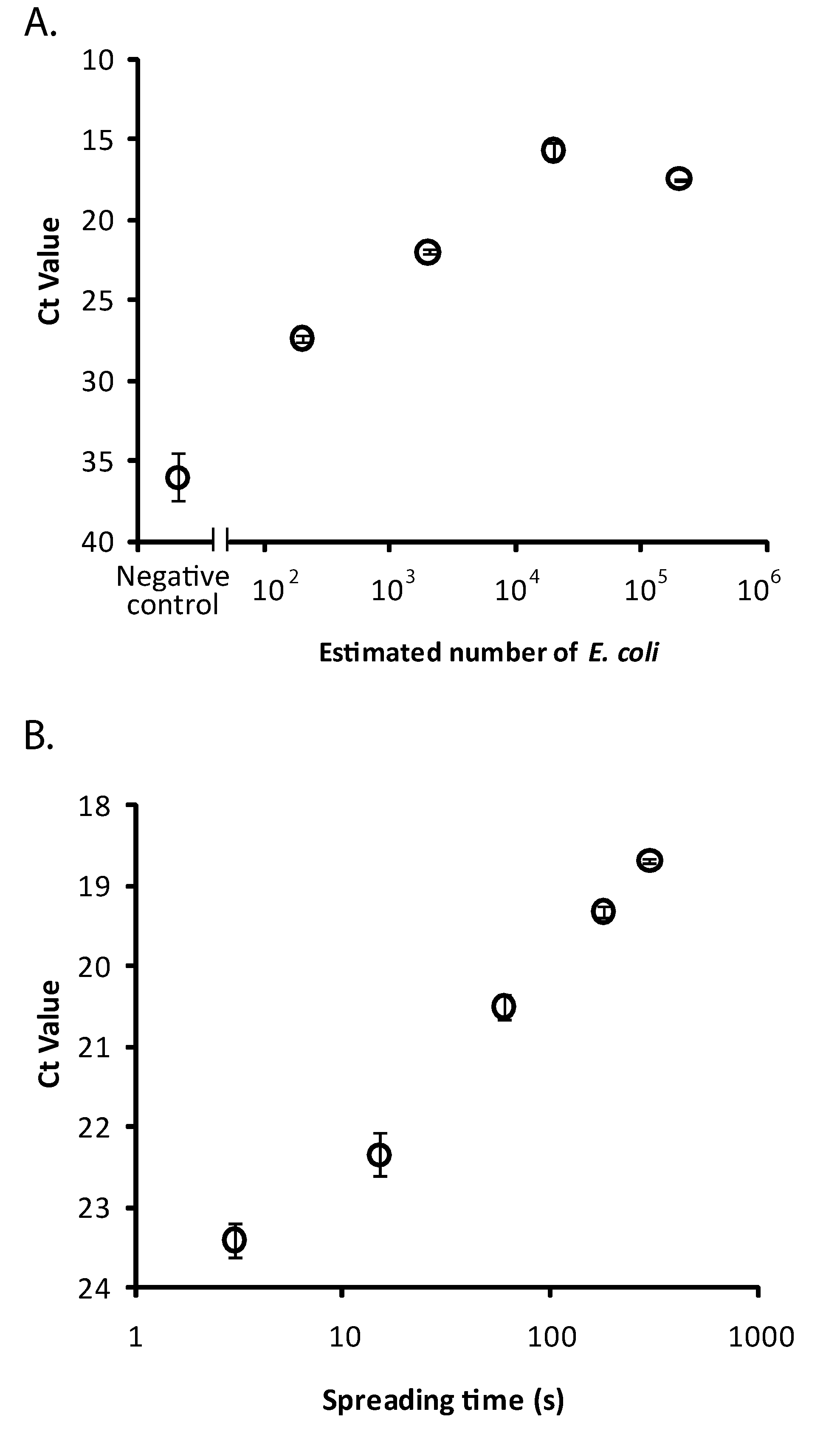

Supplement: Figure S3 — Detection of E coli genome by quantitative PCR. A) A dilution series of genomic DNA isolated from E coli was analyzed by quantitative PCR. B) Prepared samples from spreading by the ASAP air sampler detected by quantitative PCR. The negative control sample is water. The standard deviations are from triplicate samples. (TIF) [file pone.0031068.s003.tif]

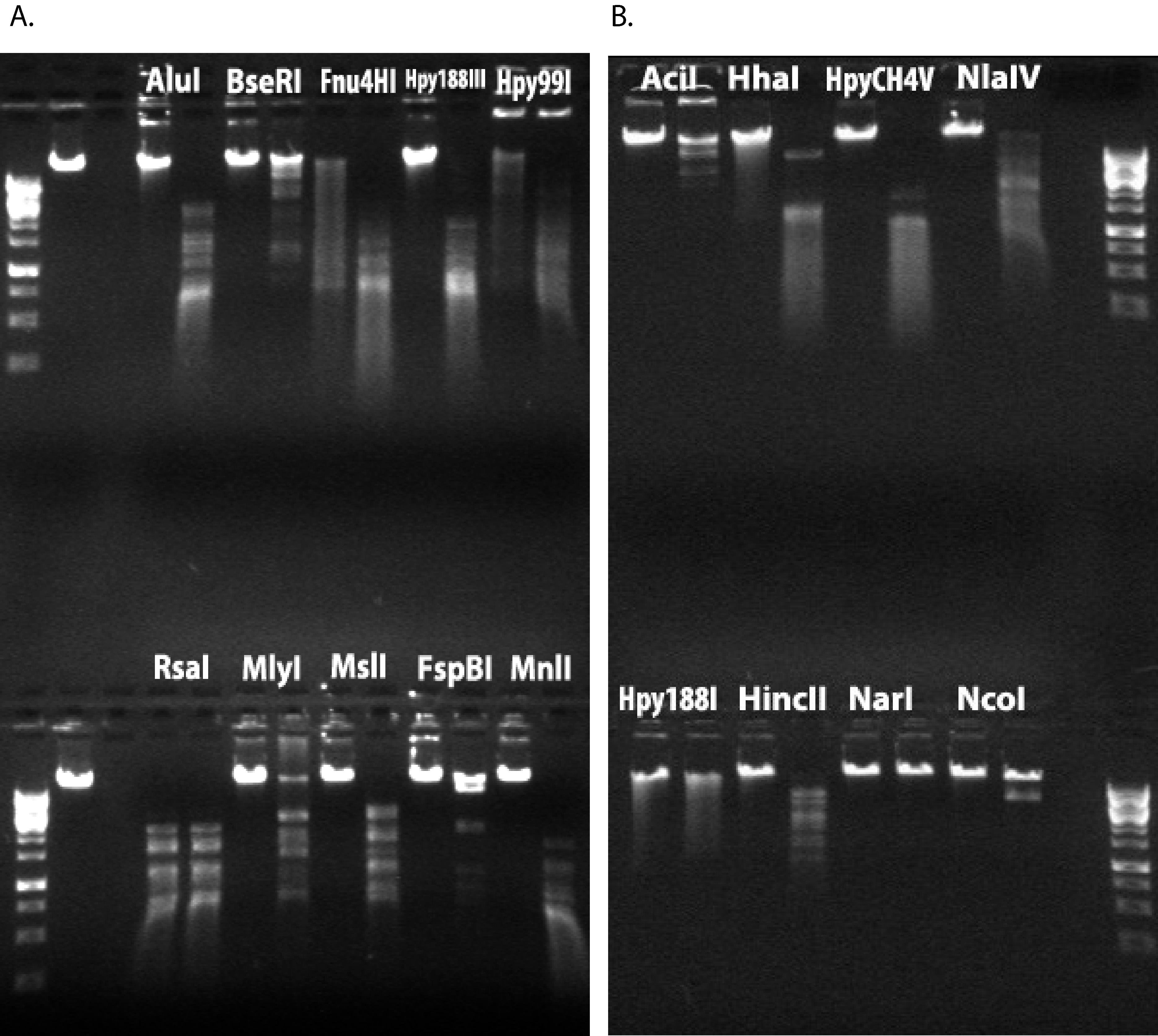

Supplement: Figure S4 — Lambda DNA Digestion products with and without preceding incubation at 65°C. For both lanes 2 in A and both lanes 10 in B: 1 kb ladder. Both lanes 3 in A: undigested Lambda DNA. Under each enzyme legend in A and B, the first lane contains DNA digested for 5 min after incubation at 65°C 5 min and the second lane contains DNA digested for 5 min after incubation at RT. (TIF) [file pone.0031068.s004.tif]

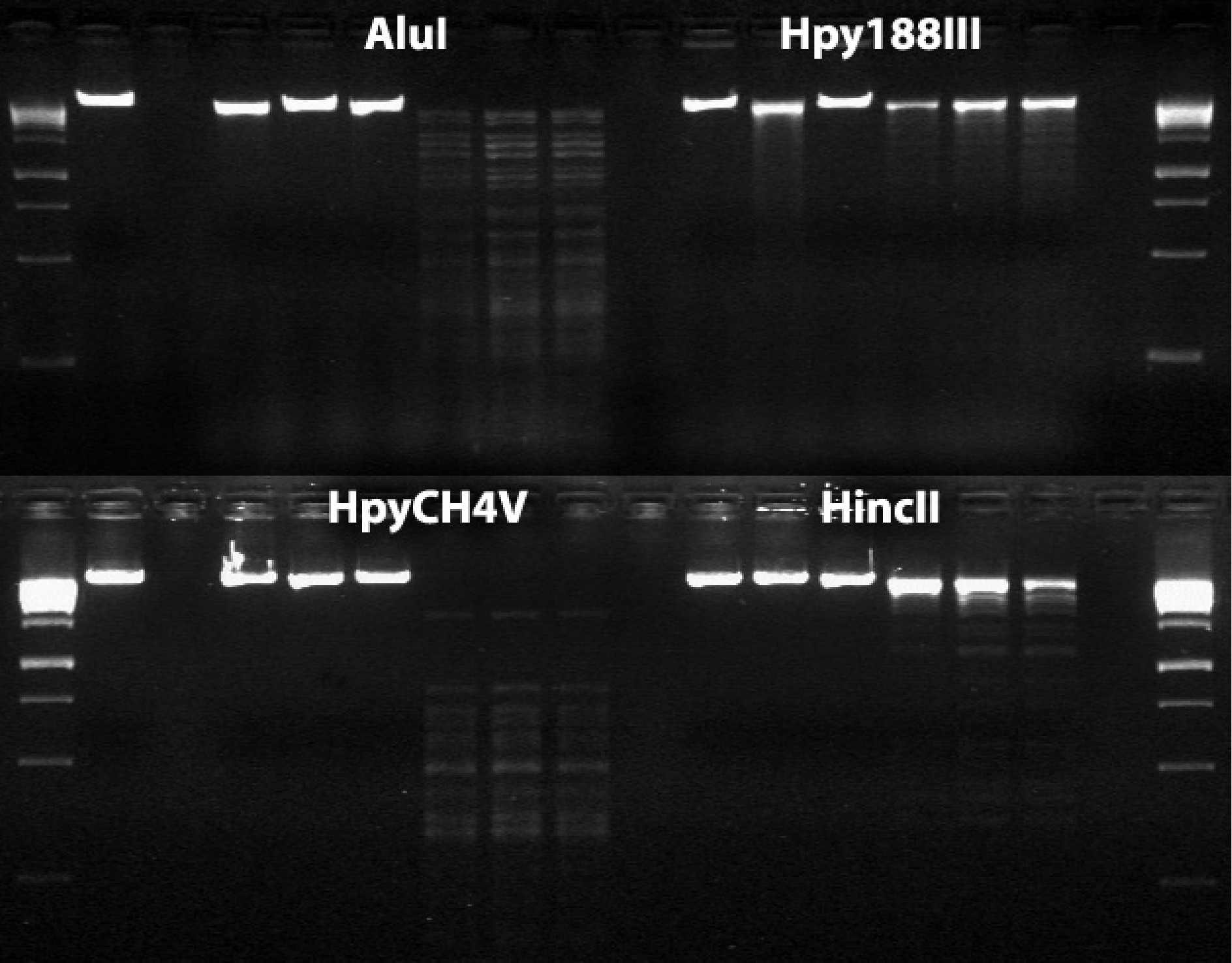

Supplement: Figure S5 — Lambda DNA Digestion products with and without preceding incubation at 65°C using different incubation periods. First and last lane in both rows: 1 kb ladder. Lane 2, both rows: undigested Lambda DNA. Under each enzyme legend, the first three lanes contain DNA digested for 5 min after incubation at 65°C for 1, 2 and 4 min respectively. The following three lanes contain DNA digested for 1, 2 and 4 min respectively, after incubation at RT. (TIF) [file pone.0031068.s005.tif]

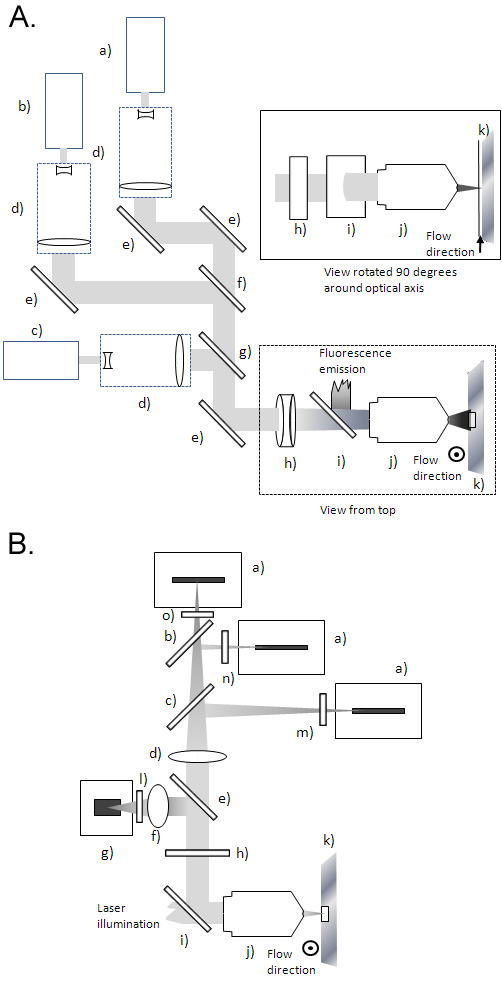

Supplement: Figure S6 — Schematic description of the optical set-up of the detection instrument. A) Optical pathway fluorescence excitation schematics. a) 640 nm laser, b) 532 nm laser, c) 491 nm laser, d) beam expenders, e) laser mirrors, f) 532/640 nm laser beam combiner, g) 491/532/640 nm laser beam combiner, h) beam shaping optics, i) triple-laser pass dichroic mirror, j) objective, and k) flow channel. B) Optical pathway fluorescence emission schematics. a) CCD line detector, b) 648 LP dichroic mirror, c) 550 LP dichroic mirror, d) tube lens, e) beam pickoff, f) beam monitoring tube lens, g) beam monitoring CCD camera, h) notch filter 532 nm, i) laser pass dichroic mirror, j) objective, k) detection channel, l) beam monitoring CCD camera filter (590/60), m)525/50 band-pass filter, n) 590/60 band-pass filter, and o) 690/60 bandpass filter. (TIF) [file pone.0031068.s006.tif]
