# Supplementary material for: Rapid Identification of Bio-Molecules Applied for Detection of Biosecurity Agents Using Rolling Circle Amplification
Source: PLoS One. 2012 Feb 22;7(2):e31068. doi: 10.1371/journal.pone.0031068 (PMC3285169; doi:10.1371/journal.pone.0031068)
Supplement: Table S1 — Observed digestion and inactivation properties of the tested restriction enzymes. (DOCX) [file pone.0031068.s007.docx]

## Table S1

|  | Digestion | Inactivation |
| --- | --- | --- |
| AluI | +^a^ | + |
| BseRI | -^b^ | + |
| Fnu4HI | + | - |
| Hpy188III | + | + |
| Hpy99I | + | - |
| RsaI | + | - |
| MlyI | - | + |
| MslI | + | + |
| FspBI | -^c^ | + |
| MnlI | + | + |
| AciI | - | + |
| HhaI | +^c^ | - |
| HpyCH4V | + | + |
| NlaIV | -^c^ | + |
| Hpy188I | - | - |
| HincII | + | + |
| NarI^d^ | - | + |
| NcoI^d^ | - | + |
| a: Complete digestion  b: Incomplete digestion  c: Slightly ambiguous band pattern  d: These enzymes were included due to misinformation of the number of cuts in Lambda | | |
