# Supplementary material for: Rapid Identification of Bio-Molecules Applied for Detection of Biosecurity Agents Using Rolling Circle Amplification
Source: PLoS One. 2012 Feb 22;7(2):e31068. doi: 10.1371/journal.pone.0031068 (PMC3285169; doi:10.1371/journal.pone.0031068)
Supplement: Text S1 — Supporting materials and methods. (DOCX) [file pone.0031068.s008.docx]

## Text S1

## Optimization of RCP digestion

To minimize the time needed for monomerization of the RCA products, the applied restriction enzyme has to digest rapidly and efficiently as well as being easily inactivated. In order to find such an enzyme, a number of candidate enzymes were chosen for further investigations. Preferred specified properties of these enzymes were high activity in different restriction enzyme buffers, blunt or short overhang cuts, 37 °C reaction temperature, 65 °C inactivation temperature and a large number of recognition sites in Lambda DNA. Since the unit definition of the enzymes refers to the amount of Lambda DNA it can digest in 1 h, it was hypothesized that an enzyme that cuts Lambda DNA frequently will have a high activity per cut and unit.

Two hundred and fifty nanograms of Lambda DNA was incubated at either 65 °C or room temperature for 5 min, then at 37 °C for 5 min in 1 × phi29 buffer (Fermentas, Burlington, Canada) and 0.5 U/µl of the respective restriction enzyme. The digestion products were analyzed by gel electrophoresis (1% agarose, 90 V) and UV visualization, as shown in Figures S4A and S4B. The tested enzymes are listed in Table S1, together with the observations made. *Rsa*I, *FspB*I and *Mnl*I were from Fermentas and *Hha*I and *Hinc*II from Pharmacia (Uppsala, Sweden). The rest of the restriction enzymes were from New England Biolabs (Ipswich, MA, USA)

Four enzymes demonstrated the desired properties and had the preferred digestion pattern. These were further analyzed with regard to their speed of digestion and inactivation, by repeating the above experiment while varying the incubation times to 1, 2 and 4 min for the inactivation and digestion respectively. Gel electrophoresis was performed with 2% agarose and 120 V for 45 min. Figure S5 clearly shows that *Alu*I and *HpyCH4*V were able to both digest and be inactivated in as short as one minute of incubation for the respective reaction. Finally, the price of the enzymes settled the matter and *Alu*I was chosen.

**Detection instrument**

Solutions containing the labeled RCPs are analyzed using a dedicated high-speed fluorescence detection instrument (Q-linea, Uppsala, Sweden). The sample solution is pushed through a flow cell with a cross section of 200 × 40 µm (WxH). The flow channels are aligned radially on a CD-format plastic disk, with appropriate optically clear lid and fluid interfaces, allowing rapid change of channel in case of a malfunction or clog. Three lasers with wavelengths of 488 nm (Calypso, 100mW, Cobolt AB, Solna, Sweden), 532 nm (Samba 300 mW, Cobolt AB, and 640 nm (Cube 640, 40 mW, Coherent Inc, Santa Clara, CA), are collimated through individually focusable beam expanders, bringing the beam diameters up to about 8 mm (1/e^2^). The beams are made collinear by a system of steerable dichroic and full mirrors (Figure S6A). The collinear beams passed through a beam shaping lens, designed to produce in conjunction with a high numerical objective (Zeiss Fluar 40x, NA 1.3, Carl Zeiss AB, Stockholm, Sweden), a line illumination profile across the interrogation volume of the flow channel. Finally, just prior to the objective entrance pupil, the laser light passes through a laser-pass dichroic mirror (Semrock Inc, Rochester, NY). Fluorescent light is emitted by RCPs pumped across the interrogation volume using a syringe pump (Tecan XLP6000, Tecan Nordic AB, Mölndal, Sweden). The emitted wavelength(s) corresponds to the emission spectra of the fluorescent labels bound to RCPs. The emitted light is collected through the objective is reflected or passes through the dichroic mirror, and is further collected by CCD line detectors (DALSA Spyder 3, 1024 pixels, line rate 5 kHz, Parameter AB, Stockholm, Sweden). Dichroic mirrors and band-pass filters are used to direct the light from each specific fluorophore to a specific detector (Figure S6B). A small portion of the fluorescent light is redirected by a beam sampler onto a CCD area detector (µEye UI-1545LE-M, Parameter AB, Stockholm, Sweden) in order to allow for channel alignment and focusing. From each detector the results of each sample run are registered as a series of x-t images where each RCP is identified through image analysis. The image analysis consists of background subtraction, pattern recognition, pattern matching across detectors for multiply fluorescent objects discrimination of RCPs and non-specific events, and RCP counting. For each reagent, a threshold for the number of RCPs is set to designate a positive sample.

The entire sequence of sample injection, image acquisition and analysis, and data processing is handled by dedicated software (Q-linea) on a PC running WinXP and equipped with two frame grabbers (DalsaCoreco X64-CL iPro, Parameter AB, Stockholm, Sweden).

**Quantitative PCR**

Water, prepared EC samples, and a dilution series of genomic EC DNA (1 fg, 10 fg, 100 fg, 1 pg, 10 pg, 100 pg, and 1 ng) was mixed with PCR mixture containing 100 nM of fwd and rev primers (GGAGTTAGCCGGTGCTTCTT and AGGCCTTCGGGTTGTAAAGT), 1 × Taq Platinum polymerase PCR buffer (Invitrogen), 2.5 mM MgCl_2_, 200 µM dNTPs, 1 × sybr green, and 30 mU/µl Taq Platinum polymerase (Invitrogen). The reaction was initiated at 95⁰C for 2 min, and cycled 45 times at 95⁰C for 15 sec and 55⁰C for 1 min in quantitative PCR equipment (MxPro Stratagene).
